# Pediatric ANCA-Associated Vasculitis: Variable Clinical Course in a Case Series of Three Patients and Literature Review

**DOI:** 10.3390/children13050712

**Published:** 2026-05-21

**Authors:** Andrei-Ioan Munteanu, Delia-Maria Nicoară, Iulius Jugănaru, Raluca Asproniu, Raluca Vasilescu, Lucian-Ioan Cristun, Otilia Mărginean

**Affiliations:** 1 Department XI Pediatrics, Discipline I Pediatrics, ‘Victor Babes’ University of Medicine and Pharmacy of Timisoara, 300041 Timisoara, Romania; andrei-ioan.munteanu@umft.ro (A.-I.M.); nicoara.delia@umft.ro (D.-M.N.); raluca.asproniu@umft.ro (R.A.); marginean.otilia@umft.ro (O.M.); 2 Department of Pediatrics I, Children’s Emergency Hospital ‘Louis Turcanu’, 300011 Timisoara, Romania; raluca.bolboase@gmail.com; 3 Research Center for Disturbances of Growth and Development in Children BELIVE, ‘Victor Babes’ University of Medicine and Pharmacy of Timisoara, 300041 Timisoara, Romania; 4 Ph.D. School Department, ‘Victor Babes’ University of Medicine and Pharmacy of Timisoara, 300041 Timisoara, Romania; lucian.cristun@umft.ro

**Keywords:** pediatrics, autoimmune diseases, vasculitis, ANCA antibodies, pathogenesis, juvenile idiopathic arthritis, granulomatosis with polyangiitis

## Abstract

**Highlights:**

**What are the main findings?**

- ANCA positivity in children can represent a spectrum of conditions, from true vasculitis with severe multi-organ involvement to alternative diagnoses such as JIA.
- Outcomes varied significantly depending on the underlying diagnosis, ranging from complete remission to chronic kidney disease.

**What are the implications of the main findings?**

- ANCA results must always be correlated with the full clinical picture to avoid misdiagnosis and inappropriate treatment.
- Individualized, phenotype-driven therapeutic strategies are essential to improve outcomes in pediatric ANCA-positive patients.

**Abstract:**

Background: Antineutrophil cytoplasmic antibody (ANCA)-associated vasculitis (AAV) represents a group of rare systemic autoimmune disorders marked by inflammation and damage to small- and medium-sized blood vessels. The clinical presentation of AAV is highly variable, ranging from isolated organ involvement to severe, life-threatening multisystem disease, posing significant challenges in diagnosis, treatment, and prognosis. Objective: To demonstrate the clinical heterogeneity and different outcomes in three pediatric cases of ANCA-positive disease and emphasize the importance of integrating clinical findings with laboratory and imaging investigations for accurate diagnosis. Methods: We present three pediatric patients (ages 12–15 years) with ANCA-positive results but distinct clinical presentations, evaluated at the Children’s Emergency Hospital “Louis Turcanu”, Timisoara, between 2020 and 2024. All cases were investigated according to EULAR/PRINTO/PReS criteria for pediatric vasculitis. Results: Case 1 (PR3-ANCA positive) developed severe multi-organ involvement, including granulomatosis with polyangiitis (GPA) with pulmonary hemorrhage, pericarditis, thrombotic events, and renal impairment, requiring intensive immunosuppression with cyclophosphamide, rituximab, and mycophenolate mofetil, ultimately developing chronic kidney disease stage 3a. Case 2 (BPI-ANCA positive) presented with purpuric lesions and painless joint swelling, responding favorably to corticosteroid therapy with subsequent remission. Case 3 (MPO-ANCA) manifested as polyarticular arthritis without other organ involvement and was ultimately diagnosed as seronegative juvenile idiopathic arthritis (JIA), achieving complete remission with adalimumab therapy. Conclusions: This case series highlights the diverse clinical and biological features of ANCA-positive conditions in children, emphasizing that ANCA positivity requires careful clinical correlation as it may indicate true vasculitis requiring aggressive treatment or alternative diagnoses such as JIA with incidental ANCA positivity. Tailored therapeutic strategies based on clinical presentation and continued research are essential to improve patient outcomes.

## 1. Introduction

Antineutrophil cytoplasmic antibodies (ANCAs) are autoantibodies directed against cytoplasmic antigens expressed in the primary granules of neutrophils and the lysosomes of monocytes. Primary neutrophil granules contain various antibacterial proteins, including lysozyme, myeloperoxidase (MPO), neutral serine proteinases (proteinase 3 [PR3], elastase, and cathepsin G), and acid hydrolases (cathepsin B and D). Autoantibodies can develop responses against any of these proteins [1].

ANCA-positive vasculitis is a group of diseases that manifest as necrotizing inflammation of small vessels with a wide range of clinical manifestations. This group includes three different clinical diseases: granulomatosis with polyangiitis (GPA), eosinophilic granulomatosis with polyangiitis (EGPA), and microscopic polyangiitis (MPA) [2]. While GPA is more common than MPA or EGPA in the general population, recent studies indicate that MPA is reported more frequently than GPA in children.

Patients who are ANCA-positive but negative for both MPO and PR3 have been reported to be positive for other types of ANCA, such as anti-BPI (bactericidal/permeability-increasing protein), cathepsin G, lactoferrin, elastase, azurocidin, and lysozyme antibodies. These are observed less frequently than MPO-ANCA and PR3-ANCA and are known as minor ANCA (x-ANCA).

In GPA, the disease affects the upper and lower respiratory tract as well as the kidneys. In MPO vasculitis, renal involvement is the most common manifestation, characterized by segmental pauci-immune necrotizing glomerulonephritis, presenting with hypertension, edema, and proteinuria. The clinical manifestations in x-ANCA vasculitis are nonspecific and, in some cases, refer to vasculitis occurring after possible infections with Gram-negative bacteria in the respiratory tract or in patients with cystic fibrosis or inflammatory bowel diseases. BPI is an antibacterial neutrophil cytoplasmic protein that plays an important role in the immune system. BPI has anti-endotoxin properties that allow it to act against Gram-negative bacterial infections [3]. It is a protein found abundantly in neutrophil azurophil granules [4,5]. This BPI protein is an important body defense mechanism against bacteria and lipopolysaccharide (LPS). Extracellular accumulation of endotoxin and BPI during invasive Gram-negative bacilli (GNB) infection occurs mainly in the tissues. Recent studies suggest that immature dendritic cells (DCs) in tissues are the target of endotoxin-rich particles coated with BPI [6]. The N-terminus of BPI binds with high affinity to LPS and is responsible for its endotoxin-neutralizing activity, while the C-terminus has a role in bacterial opsonization.

BPI-ANCA has been observed to be positive in vasculitis, cystic fibrosis [4], inflammatory bowel disease (IBD), sclerosing cholangitis [7], diffuse panbronchiolitis (DPB), bronchiectasis, autoimmune hepatitis, primary sclerosing cholangitis, and Felty’s syndrome [8]. In 1998, Kobayashi described the high frequency of detection of BPI-ANCA in chronic airway infections (e.g., diffuse panbronchiolitis and bronchiectasis) and postulated that BPI-ANCA would suppress BPI activity and thus suppress neutrophil activity in Gram-negative bacilli infections [9]. One study observed a positive association between serum titer of BPI-ANCA and disease activity. It is possible that BPI-ANCA may act as one of the persistent stimulatory agents to produce inflammatory cytokines [10]. By decreasing antimicrobial function, BPI-ANCA may contribute to decreased bacterial clearance and, thus, to the maintenance of inflammation in some cases in the lungs [11].

However, it is not clear whether anti-BPI ANCAs are a cause or an effect of infections. If the macrophage functions as an antigen-presenting cell in a local environment with a lipopolysaccharide adjuvant, tolerance may be lost, producing anti-BPI ANCA [12]. These phenomena indicate that anti-BPI ANCA may be produced during chronic inflammatory diseases through the following mechanisms:

(1) Anti-BPI ANCAs bind to circulating BPI overexpressed by infection, inducing immune complex (IC) formation.
(2) Circulating immune complexes can bind to neutrophils with overexpressed BPI.
(3) TNF-α facilitates signaling mediated by immune complexes, such as spleen tyrosine kinase (Syk), leading to neutrophil extracellular trap (NET) formation.
(4) These processes contribute to the development of systemic vasculitis [13]. Although it is known that BPI-ANCA are involved in the pathogenesis of systemic vasculitis, it remains unclear whether they play a pathogenic role in the development of vasculitis.

## 2. Materials and Methods

This retrospective case series presents three pediatric patients with ANCA-positive serology evaluated at the 1st Department of Pediatrics, Children’s Emergency Hospital “Louis Turcanu”, Timisoara, Romania, between 2020 and 2024. All patients were investigated according to the diagnostic criteria for ANCA-associated vasculitis (AAV) established by the European Alliance of Rheumatology (EULAR), Pediatric Rheumatology International Trials Organization (PRINTO), and European Society for Pediatric Rheumatology (PReS). Inclusion criteria comprised the following: (1) age below 18 years at diagnosis; (2) positive ANCA serology confirmed by at least two independent laboratory determinations; (3) clinical evaluation performed at our center with complete diagnostic workup; and (4) minimum follow-up of three months from initial presentation. All ANCA-positive pediatric patients seen at our institution during the study period who fulfilled these criteria were included; no selective case reporting was performed. The three cases presented represent the totality of ANCA-positive pediatric patients evaluated at our center during the defined study interval (2020–2024). The follow-up duration ranged from 12 to 48 months (median 24 months). Diagnostic workup included complete blood count (CBC), inflammatory markers (C-reactive protein [CRP], erythrocyte sedimentation rate [ESR], procalcitonin, ferritin), renal function tests, urinalysis with 24 h protein quantification, immunological panel (antinuclear antibodies [ANA], rheumatoid factor [RF], circulating immune complexes, complement levels, ANCA antibodies with MPO, PR3, and BPI specificity), and infectious disease screening (Hepatitis B and C, HIV, cytomegalovirus, Toxoplasma gondii, Epstein–Barr virus, Borrelia, and Mycoplasma). Imaging studies included chest computed tomography (CT), echocardiography, and abdominal ultrasound as clinically indicated. Tissue biopsies were performed when necessary for diagnostic confirmation. ANCA testing was performed using a two-step methodology: initial screening by indirect immunofluorescence (IIF) on ethanol-fixed neutrophils to determine the staining pattern (cytoplasmic [c-ANCA] or perinuclear [p-ANCA]), followed by antigen-specific confirmation using enzyme-linked immunosorbent assay (ELISA) for anti-PR3, anti-MPO, and anti-BPI antibodies. This combined IIF plus ELISA approach is in accordance with the 2017 international consensus on ANCA testing and recommended guidelines for the diagnosis of ANCA-associated vasculitis. Positivity was defined as a titer exceeding the manufacturer’s recommended cut-off on at least two separate determinations performed at our institution’s accredited immunology laboratory.

Treatment protocols followed EULAR recommendations for pediatric vasculitis, with modification based on individual clinical response and disease severity. Clinical and laboratory monitoring was performed regularly throughout the treatment period and follow-up. Informed consent for data use was obtained, and the study was approved by the Ethics Committee for Research of the “Victor Babes” University of Medicine and Pharmacy, Timisoara (approval number 12241/3 July 2024).

## 3. Results

We present three cases of ANCA-positive conditions to explore and analyze their diverse patterns of onset, clinical and biological progression, and response to treatment.

### 3.1. Case 1: PR3-ANCA-Positive Vasculitis (Granulomatosis with Polyangiitis)

#### 3.1.1. Clinical History

Patient C.A., a 15-year-old female, presented to the clinic with dysuria and sinusitis manifested by fronto-parietal headache accompanied by muco-bloody nasal secretions that did not respond to oral antibiotic treatment (second-generation cephalosporin) for 7 days. On the 10th day, she developed migratory arthralgias of the shoulders, coxo-femoral joints, knees, and ankles bilaterally, accompanied by functional impairment. Biological investigation revealed an inflammatory syndrome (ESR = 57 mm/h, CRP = 11 mg/dL). ENT symptomatology persisted, and the arthritis extended to the fingers bilaterally. Re-evaluation after approximately two weeks showed worsening of the general condition. An autoimmune pathology, possibly vasculitic, was suspected. Serial biological investigations demonstrated progressive inflammation (Table 1). A chronological summary of the clinical course is provided below: Week 0–2: onset with sinusitis, dysuria, and migratory arthralgia; Week 2–4: diagnosis of PR3-ANCA-positive GPA, initiation of prednisone 1 mg/kg/day and methotrexate 15 mg/week subcutaneous; Week 4–6: acrocyanosis, Doppler-confirmed basilar vein thrombosis, addition of rivaroxaban, cardiac arrhythmias with increased pericardial effusion; Week 6–8: escalation to intravenous cyclophosphamide 15 mg/kg (cycles I–VI) and methylprednisolone pulse therapy, psychiatric episode suspected as corticosteroid-induced psychosis; Week 12: hemorrhagic cystitis after cycle IV cyclophosphamide, spontaneous resolution; Week 16: pulmonary hemorrhage confirmed by chest CT and bronchoscopy after cycle VI cyclophosphamide; Week 16–20: intensive care management, supportive treatment, decision not to perform plasmapheresis (parental refusal); Month 5–9: rituximab 375 mg/m^2^/week (four cycles), initial improvement followed by clinical and renal deterioration; Month 9: intravenous immunoglobulin 2 g/kg (immunosuppressive dose); then, we continued with mycophenolate mofetil 2 g/day with tapering prednisone. Maintenance rituximab at month 14 and month 20, achieving clinical and biological remission with stabilized chronic kidney disease stage 3a.

#### 3.1.2. Diagnosis

**Figure 1:**
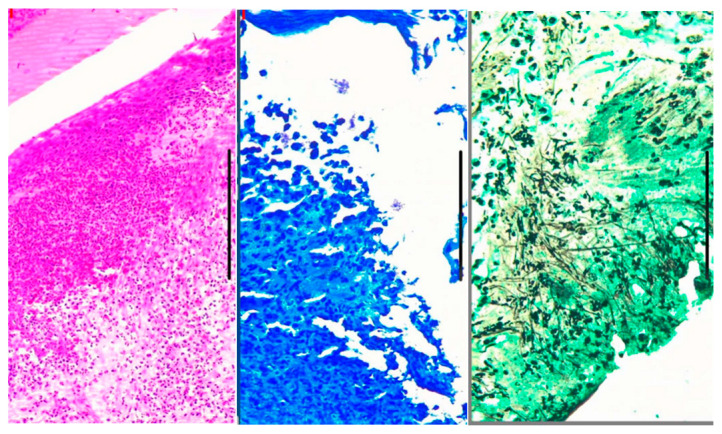
Nasal biopsy: Giemsa, PAS, and Grocott staining, cocci arranged in clusters, and isolated hyphae (Candida albicans and Staphylococcus aureus).

At this point, clinical and biological arguments supported a diagnosis of PR3-ANCA-positive vasculitis (granulomatosis with polyangiitis). A nasal mucosal biopsy was performed for diagnostic confirmation, revealing inflammatory cells in the blood vessels and nasal mucosa, along with an important infiltrate with clustered and isolated hyphae (Candida albicans and Staphylococcus aureus identified on Giemsa, PAS, and Grocott) (Figure 1).

#### 3.1.3. Treatment and Evolution

**Figure 2:**
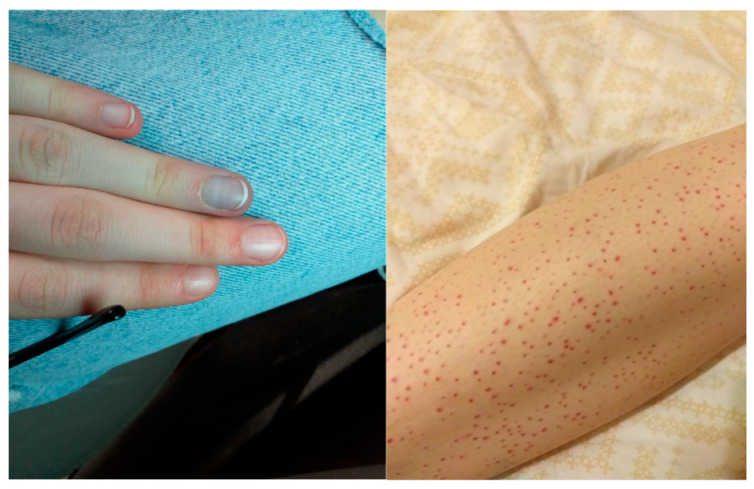
Purpuric vasculitic lesions of the lower limbs and acrocyanosis with ischemic discoloration of the IV and V fingers bilaterally in Case 1 (PR3-ANCA-positive GPA), occurring approximately two weeks after diagnosis and corresponding to thrombotic occlusion of the basilar vein confirmed by Doppler ultrasonography.

Bone marrow aspiration, chest CT, and cardiological evaluation were also performed, revealing a small to moderate amount of pericardial effusion. The diagnosis of systemic PR3-ANCA-positive vasculitis (granulomatosis with polyangiitis) was established, and treatment was initiated according to EULAR recommendations with oral prednisone 1 mg/kg/day together with disease-modifying antirheumatic drugs (DMARDs): subcutaneous methotrexate 15 mg/week. Approximately two weeks after diagnosis, the patient developed acrocyanosis of fingers III, IV, and V of the upper limbs with paresthesia/pain sensation in bilateral hands (Figure 2).

Doppler ultrasonography revealed complete thrombosis of the basilar vein in the forearm and elbow. A positive thrombophilia panel was identified for heterozygous Factor V Leiden, MTHFR C677T, PAI-1 4G/5G, and EPCR A2/A3. Treatment with oral rivaroxaban 20 mg/day was initiated. Despite the complex therapy applied, the patient’s condition clinically deteriorated with repeated cardiac arrhythmias. Cardiologic examination revealed bradycardia (HR = 40–50 beats/min) and increased pericardial fluid (approximately 1.6 cm). Biologically, there was an increasing inflammatory syndrome (Table 1).

**Figure 3:**
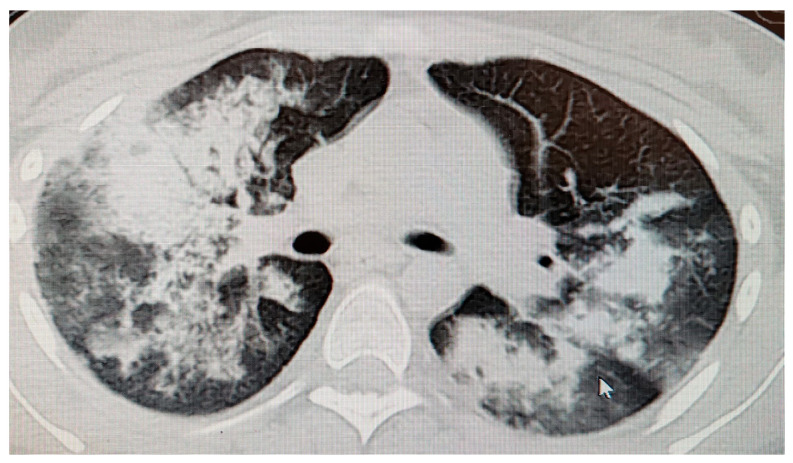
Chest computed tomography (CT) demonstrates bilateral ground-glass opacities with a perihilar predominance and areas of consolidation in the lower lobes, consistent with diffuse alveolar hemorrhage in the context of AAV-associated pulmonary hemorrhage. Case 1 (PR3-ANCA-positive GPA), 15-year-old female, after cycle VI of cyclophosphamide therapy.

Given the systemic involvement with progression of renal impairment, it was decided to escalate therapy with the initiation of intravenous cyclophosphamide 15 mg/kg (Endoxan) and methylprednisolone pulse therapy 500 mg/day for 3 days. Oral prednisone 80 mg/day was continued. Approximately seven days after the pulse therapy, the patient presented with neuropsychiatric disorders (bradykinesia, uncoordinated movements, psychomotor agitation, impulsivity with heteroaggression, and hallucinations). A psychotic episode due to high-dose corticosteroids was suspected, and psychiatric treatment was instituted. After cycle IV of cyclophosphamide, hemorrhagic cystitis was diagnosed despite the administration of Mesna (Uromitexan), which remitted spontaneously with supportive treatment within 3 days. After cycle VI of cyclophosphamide, the patient presented with a pre-syncopal episode, altered general condition, and acute respiratory dysfunction (SaO2 80–83% in room air, BP 119/70 mmHg). Biologically, there was an increase in inflammatory markers (Table 1). Chest CT (Figure 3) and bronchoscopy were performed, confirming the diagnosis of pulmonary hemorrhage.

The possibility of performing plasmapheresis was discussed, but parental consent was not obtained, and the patient had a slow, favorable evolution with supportive treatment in the intensive care unit. Although plasmapheresis (plasma exchange) is recommended in cases of severe AAV with pulmonary hemorrhage and/or rapidly progressive glomerulonephritis, recent evidence from the PEXIVAS trial has demonstrated that plasma exchange does not reduce the risk of end-stage kidney disease or death in AAV when added to standard immunosuppression. Given parental refusal and the gradual clinical stabilization observed, a conservative approach with intensified supportive care was pursued. After remission of the pulmonary hemorrhage episode, immunosuppressive treatment with rituximab 375 mg/m^2^/week was initiated, with initial clinical improvement. The decision to initiate rituximab despite prior infectious complications (hemorrhagic cystitis, Candida albicans, and Staphylococcus aureus identified on nasal biopsy) was taken after multidisciplinary discussion. Cyclophosphamide was discontinued due to cumulative toxicity (hemorrhagic cystitis) and persistent disease activity, and rituximab was selected based on evidence of non-inferiority to cyclophosphamide for induction of remission in ANCA-associated vasculitis (RITUXVAS and RAVE trials). Active fungal colonization was treated with antifungal therapy prior to and during rituximab initiation, and infectious parameters were monitored closely throughout. After the fourth cycle of rituximab, the patient’s condition worsened with increased inflammatory markers and renal function alteration (Table 1). Ultrasound showed small amounts of ascites and pericardial effusion, and Doppler ultrasonography revealed total thrombosis of both internal saphenous veins, necessitating an increase in the dose of enoxaparin initiated during the pulmonary hemorrhage episode.

In the following period, the patient was monitored weekly without clinical or laboratory improvement, leading to the decision to start treatment with intravenous immunoglobulin at an immunosuppressive dose of 2 g/kg. Subsequently, treatment was continued with oral prednisone in tapering doses and mycophenolate mofetil 2 g/day, with gradual clinical improvement. At 4 months, the maintenance dose of rituximab was administered, and the second cycle at a 6-month interval was given to sustain clinical response. Daily treatment continued with mycophenolate mofetil and prednisone 5 mg/day, under which the patient achieved clinical and biological remission.

Renal function stabilized with a glomerular filtration rate (GFR) of 48–52 mL/min/1.73 m^2^, corresponding to chronic kidney disease stage 3a, with proteinuria of 1.8 g/24 h in a volume of 3000 mL/24 h. ANCA-PR3 level was 7.2 U/mL (normal value < 3 U/mL). The patient’s general condition remained good at subsequent evaluations, but an increase in TSH to 8.08 µIU/mL was noted, and two “cold” nodules were observed ultrasonographically and scintigraphically. Endocrinologic substitution treatment with levothyroxine was initiated. At the last evaluation, the patient had no clinical signs of disease activity and no inflammation, but a gradual increase in nitrogen retention parameters was observed. She was proposed for renal biopsy and nephrologic monitoring.

### 3.2. Case 2: BPI-ANCA-Positive Vasculitis

#### 3.2.1. Clinical History

**Figure 4:**
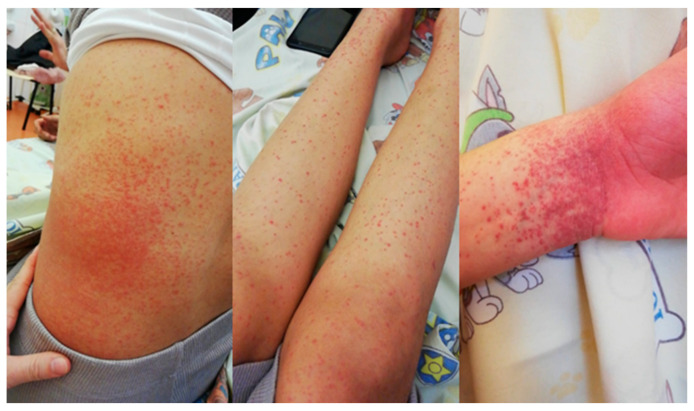
Extensive palpable purpuric lesions distributed across the lower extremities, with a characteristic tendency toward coalescence, associated with bilateral knee swelling. Lesions are non-blanching and consistent with small vessel vasculitis. Case 2 (BPI-ANCA positive), 12-year-old female, at initial presentation.

Patient B.N., a 12-year-old female, presented to our clinic with purpuric lesions of the lower limbs, left radiocarpal joint, and left abdominal flank (Figure 4), which progressively spread to the thighs and arms, accompanied by painless swelling of the large joints. Considering the systemic nature of the lesions, comprehensive biological investigations were performed.

#### 3.2.2. Laboratory Investigations and Imaging

Laboratory findings revealed normal complete blood count (CBC), no elevations of inflammatory markers (CRP, ESR, procalcitonin, and ferritin), normal immunoglobulin levels (IgA, IgM, IgG), no coagulation disorders, and no changes in 24 h urine analysis. A slight decrease in complement C4 level was observed (0.08 g/L; normal range: 0.1–0.4 g/L), raising suspicion for systemic immune pathology. Immunological workup showed negative antinuclear antibodies (ANA) and rheumatoid factor (RF), inconclusive circulating immune complexes, and positive ANCA antibodies specific for the BPI fraction.

Infectious etiologies including Hepatitis B, C, HIV, cytomegalovirus, Toxoplasma gondii, Epstein–Barr virus, Borrelia, and Mycoplasma were excluded through serological testing. Nasopharyngeal, urine, and blood cultures were performed without detecting a possible infectious focus. Ear, nose, throat (ENT) and ophthalmologic consultations were performed without revealing suspicious lesions. Chest computed tomography (CT) showed no pulmonary changes.

#### 3.2.3. Treatment and Evolution

During a seven-day hospitalization, empirical broad-spectrum antibiotics (third-generation cephalosporins) and antihistamines were administered without clinical improvement. Oral prednisone 1 mg/kg/day with gradual tapering over three months was initiated at discharge. Complete remission of purpuric lesions was achieved at day 21 of treatment.

At four-month re-evaluation, ANCA serology was negative. Over a 48-month follow-up period, recurrent self-limited purpuric episodes were documented exclusively in association with upper respiratory tract infections, each resolving spontaneously without additional intervention. No systemic organ damage occurred throughout the observation period.

This case is broadly consistent with the theoretically described mechanisms of BPI-ANCA vasculitis; however, definitive confirmation is limited by the absence of tissue biopsy, representing a recognized diagnostic limitation. The mild, self-limited clinical course with normal inflammatory markers at baseline, spontaneous ANCA negativization, and absence of disease progression over 48 months are collectively more consistent with a post-infectious immune phenomenon than with primary systemic vasculitis.

### 3.3. Case 3: MPO-ANCA Positive, Diagnosed as Juvenile Idiopathic Arthritis

#### 3.3.1. Clinical History and Laboratory Investigations

Patient M.A., a 13-year-old female, presented to the clinic with swelling of the right-hand radiocarpal joint, later associated with arthritis of the left knee and right ankle bilaterally, accompanied by morning stiffness lasting about 30 min and functional impairment. Clinical examination and laboratory investigations revealed systemic inflammation (ESR = 80 mm/h, D-dimer 1200 ng/mL, fibrinogen 471 mg/dL, CRP = 75 mg/L), normocytic anemia (Hb = 9.9 g/dL), elevated IgG (20.59 g/L), elevated IgE (4.78 g/L), with no changes in nitrogen retention parameters, no proteinuria, and no hematuria. Native radiocarpal magnetic resonance imaging (MRI) depicted significant radiocarpal and thumb osteoarthritis with moderate fluid accumulation in the carpal bones and extensor tendons. Considering the onset of strictly articular complaints over 6 weeks in a child under 16 years of age, autoimmune markers were investigated: antinuclear antibodies (ANA) were negative, HLA-B27 was negative, and anti-cyclic citrullinated peptide (CCP) antibodies were negative. From the patient’s history, repeated episodes of sinusitis, sometimes accompanied by nasal bleeding, were noted, for which ANCA testing was performed with a positive result for anti-MPO (myeloperoxidase) at a titer of 1:320. The diagnosis of ANCA-MPO vasculitis was suspected, and further investigations were continued: ENT, ophthalmologic, chest CT, cardiologic, nephrology, and abdominal ultrasound evaluations were performed, revealing no pathologic lesions beyond the articular involvement.

#### 3.3.2. Treatment and Evolution

Subcutaneous methotrexate 15 mg/m^2^/week was initiated alongside bridging oral prednisone 1 mg/kg/day, tapered over three months. At the 3-month evaluation, complete clinical and biological remission was confirmed: no synovitis, no vasculitic features, normalization of inflammatory markers (ESR 13 mm/h, CRP 1 mg/L), and no elevation in nitrogen retention parameters. However, at month 6, disease relapse was documented, with an increase in inflammatory markers (ESR 32–55 mm/h, CRP 18 mg/L) and clinical recurrence of polyarticular arthritis, despite ongoing DMARD therapy.

#### 3.3.3. Diagnostic Considerations

A diagnostic challenge arose in classification between seronegative juvenile idiopathic arthritis (JIA) polyarticular form or systemic MPO-ANCA-positive vasculitis with only articular manifestations. The patient was investigated clinically, biologically, and with imaging for diagnostic support without detecting any other organ involvement. Nasal and cutaneous biopsy for the suspicion of small vessel vasculitis was proposed, but the parents refused. The key clinical arguments that allowed us to ultimately favor a JIA diagnosis over MPO-ANCA vasculitis were the following: (1) strictly articular presentation without any evidence of systemic vasculitic manifestations (no renal involvement, no pulmonary findings, no cutaneous vasculitis, and no neuropathy) despite comprehensive multi-organ evaluation; (2) normal renal function with absence of proteinuria and hematuria throughout the disease course, inconsistent with MPO-ANCA-associated glomerulonephritis; (3) clinical and biological response to adalimumab, a biologic therapy targeting TNF-α that does not have a recognized role in treating AAV but is a first-line biologic for polyarticular JIA; (4) ANCA-MPO negativization following adalimumab therapy, suggesting that positivity may have reflected a reactive or nonspecific immune phenomenon rather than a driving pathogenic mechanism. The distinction between MPO-ANCA vasculitis and JIA in this case was further supported by the 2006 EULAR/PReS classification criteria for pediatric AAV, which require evidence of at least one of the following: renal involvement, pulmonary hemorrhage, granulomatous inflammation on biopsy, or peripheral neuropathy—none of which were present in our patient. We acknowledge that the MPO positivity at a high titer (1:320) and the history of recurrent sinusitis with epistaxis raised legitimate concern for early or limited AAV; however, the overall clinical trajectory and therapeutic response were more consistent with JIA. A chronological summary of Case 3: Month 0: onset of polyarticular arthritis (right wrist, left knee, right ankle); Month 1–2: diagnosis workup, MPO-ANCA confirmed positive (1:320) by two independent laboratories, ENT/renal/pulmonary evaluation negative; Month 2: initiation of methotrexate 15 mg/m^2^/week + prednisone bridging therapy; Month 5: initial remission (ESR 13 mm/h, CRP 1 mg/L); Month 6: relapse with polyarticular flare, escalation to adalimumab 40 mg/2 weeks; Month 7: complete clinical remission; Month 9: ANCA-MPO negative; Months 9–24: sustained remission on Adalimumab monotherapy.

### 3.4. Clinical Course and Therapeutic Management Across the Three Cases 

Table 2 summarizes three cases exemplifying varying presentations of AAV.

## 4. Discussion

Although several hypotheses have been proposed, the exact pathogenesis of ANCA-associated vasculitis (AAV) remains unclear. PR3-ANCA is typically associated with granulomatous vasculitis, whereas MPO-ANCA correlates with necrotizing small vessel vasculitis. Granulomas are not a feature of MPO-ANCA-associated vasculitis [14]. In general, PR3-ANCA is associated with GPA, whereas MPO-ANCA is predominantly found in MPA; however, there is overlap, and MPO-ANCA-positive GPA and PR3-ANCA-positive MPA are the focus of recent research. Cases with dual seropositivity for PR3-ANCA and MPO-ANCA are rare [15].

ANCA-positive conditions in pediatric patients represent severe, potentially life-threatening disorders that require early diagnosis, aggressive treatment when indicated, and prolonged monitoring. However, as demonstrated by our three cases, ANCA positivity does not always indicate true vasculitis and requires careful clinical correlation. The three cases presented illustrate the remarkable heterogeneity of ANCA-positive conditions in children. Case 1 (PR3-ANCA positive) exemplified the severe, life-threatening potential of granulomatosis with polyangiitis, with multiple organ involvement including pulmonary hemorrhage, pericarditis, thrombotic complications, and progressive renal impairment, necessitating aggressive immunosuppression with cyclophosphamide, rituximab, and mycophenolate mofetil, ultimately resulting in chronic kidney disease stage 3a. Case 2 (BPI-ANCA positive) demonstrated a relatively benign, self-limited course with favorable response to corticosteroids and subsequent remission, though requiring vigilant monitoring for potential systemic complications. Case 3 (MPO-ANCA positive) presented diagnostic challenges, ultimately being classified as seronegative juvenile idiopathic arthritis with incidental ANCA positivity, responding favorably to biological therapy with adalimumab.

The pathogenesis of AAV is multifactorial, involving genetic susceptibility, environmental triggers, and immune dysregulation [15]. ANCA-mediated neutrophil and monocyte activation—triggered by surface expression of PR3 and MPO in response to proinflammatory cytokines—initiates small vessel injury through signal transduction cascades. This mechanism directly underlies the severe multi-organ involvement observed in Case 1.

Genome-wide association studies have identified 33 susceptibility variants, predominantly in the MHC region; SERPINA1 (encoding alpha-1-antitrypsin, the principal PR3 inhibitor) and PRTN3 are specifically linked to PR3-ANCA disease, while MPO-ANCA associates with HLA-DQ [16,17]. These genetic differences likely contribute to the more aggressive granulomatous phenotype characteristic of PR3-positive patients such as Case 1.

T-cell dysregulation, complement activation via C5a, and HLA-linked susceptibility further modulate disease severity: PR3-ANCA associates with DPB1/HLA-DRB4 and MPO-ANCA with HLA-DQ [18,19,20,21]. These immunogenetic differences partly explain the contrasting phenotypic severity observed between our PR3-positive Case 1 and the MPO-positive Case 3.

Infection-triggered ANCA generation via molecular mimicry, epigenetic changes, and NET formation is directly relevant to Cases 1 and 2, where respiratory pathogens likely acted as disease initiators [7,22,23]. Persistent NET-mediated antigen exposure may sustain ANCA production and perpetuate the inflammatory cycle, consistent with the protracted course observed in Case 2.

BPI exerts anti-endotoxic properties that allow it to act against Gram-negative bacterial infections (GNB) [3]. Anti-BPI ANCAs bind to excess circulating BPI, which is exposed following infections, leading to the formation of immune complexes (IC). Circulating immune complexes can bind to neutrophils with excessive BPI expression. TNF-α facilitates IC-mediated signaling, such as spleen tyrosine kinase (Syk), leading to the formation of neutrophil extracellular traps (NETs), which contribute to the development of systemic vasculitis [13]. Additionally, BPI delivers components of GNB to dendritic cells for processing [6]. However, we emphasize that the literature reports that the role of BPI-ANCA remains unclear, and it is not definitively established whether they are a cause or consequence of infection and inflammation [6,13].

Unlike the BPI-ANCA mechanism described above, our case of PR3-ANCA-positive vasculitis (Case 1) aligns well with reports in the specialized literature regarding the onset of symptoms, with initial upper respiratory tract involvement [24], ENT manifestations, and shortly thereafter joint and small vessel involvement. The upper respiratory tract is involved in 70–100% of cases. Otologic involvement may be the first and only sign of GPA; it has been reported that 25% of patients with GPA presented with serous otitis media, and 6% of patients presented with hearing loss as the initial sign of the disease [25]. In an extensive study of children with GPA in Canada, 43% of those with multisystem disease had ophthalmic complications, while in a European study, this rate was approximately half at only 21% [26]. On the infectious side, chronic nasal colonization with Staphylococcus aureus is associated with an increased risk of relapse in patients with an established diagnosis of GPA [27]. This infection, which we also detected in our Case 1, proved difficult to treat, becoming multi-resistant to treatment and maintaining inflammation in the sinuses, producing purulent secretions that were difficult to control in the context of drug-induced immunosuppression.

In 2005, the vasculitis working group of the European Society of Pediatric Rheumatology (PRES), supported by the European League Against Rheumatism (EULAR), proposed new pediatric-specific classification criteria for GPA/MPA [28,29]. In an international cohort study that included 105 patients with childhood-onset ANCA-associated vasculitis, evidence of active disease was still present in more than 60% of patients 12 months after diagnosis [30]. This is confirmed in our Case 1, where at 12 months, the vasculitis was not in remission. Almost 6 months after obtaining inactive disease, we observed sometimes significant variations in renal function at the level of nitrogen retention, despite clinical and inflammatory disease inactivity. This leads us to consider what was observed in a large population-based cohort study, i.e., that renal lesions were the most common complication, with significant rates of end-stage renal disease reported after long-term follow-up [31].

Beyond renal involvement, Case 1 also experienced one of the most life-threatening complications of AAV-pulmonary hemorrhage, which, for some, may pose problems of differential diagnosis and therapeutic management, especially in intensive care units where there is limited experience with plasmapheresis. Studies certify that pulmonary manifestations are frequent, with pulmonary hemorrhage being the most concerning [32,33]. The mortality rate in pulmonary hemorrhage (PH) associated with AAV has been reported to range from 18% to 50% within 1 year, even with adequate treatment or mechanical ventilation.

Beyond the classic triad, AAV may involve virtually any organ system. Atypical manifestations reported in the literature include acute pancreatitis, cystic lung disease, splenic infarctions, and CNS involvement (hypertrophic pachymeningitis, PRES, cerebrovascular events, or hypophysitis) [34,35]. This broad potential for systemic damage underscores the need for comprehensive multi-organ evaluation at diagnosis, as performed in all three of our cases. Considering the above, we report that it was difficult to support the diagnosis of corticosteroid-induced psychosis in our Case 1 without having clear imaging evidence that we were not dealing with vasculitic neurological manifestations. Imaging at that time was not performed due to alterations in renal function and concerns about contrast-induced nephropathy.

Compared with pulmonary hemorrhage, venous thromboembolism (VTE) is poorly described in the literature. The extensive venous thromboses encountered in Case 1, superimposed on neurological manifestations and in combination with pulmonary hemorrhage, posed major management problems. The coagulation/anticoagulation balance is often difficult to define, and the mortality rate for VTE is approximately 0–15.4%, with most cases dying from right-sided heart failure related to fulminant pulmonary embolism (PE) [36].

Another rare complication encountered in Case 1 is cardiac involvement in GPA. This is rare and potentially fatal; the European Vasculitis Study Group reported an incidence of 5.7% in 535 patients newly diagnosed with GPA, microscopic polyangiitis (MPA), or limited vasculitis. Another Norwegian study reported an incidence of 20%, concluding that these patients are at increased risk of initial treatment resistance and disease relapse. Similarly, the French Vasculitis Study Group found that cardiac involvement was a risk factor for poor overall prognosis and increased relapses [37]. In our Case 1, pericarditis at onset forced us to administer high doses of corticosteroids in pulse therapy, which led to the control of cardiac symptoms but to neurological decompensation.

Based on the diagnostic challenges highlighted by our three cases, we propose the following stepwise diagnostic approach for the pediatric clinician encountering a child with unexplained ANCA positivity: Step 1—Confirm ANCA positivity using both IIF and antigen-specific ELISA (PR3, MPO, BPI); a positive result on IIF alone is insufficient. Step 2—Perform systematic multi-organ assessment, including renal function (urinalysis with microscopy, 24 h proteinuria, serum creatinine), pulmonary evaluation (chest CT, spirometry if age-appropriate), ENT examination, ophthalmologic assessment, cardiac evaluation (echocardiography), and peripheral nervous system evaluation. Step 3—Evaluate the overall clinical picture: if systemic vasculitis criteria are met (EULAR/PRINTO/PReS 2006 criteria), initiate immunosuppressive therapy according to disease severity. Step 4—If organ involvement is absent or equivocal, consider alternative diagnoses including JIA, IBD-associated ANCA positivity, or post-infectious reactive immune phenomena. Step 5—Obtain tissue biopsy when feasible; its absence significantly limits diagnostic certainty and should be documented explicitly. Step 6—Monitor ANCA titers serially; their evolution (rising, stable, or falling) provides important prognostic and therapeutic information. This algorithm is not intended to replace multidisciplinary clinical judgment but to provide a structured framework for initial assessment that may reduce the risk of misclassification and inappropriate therapy.

Case 2 represents the mildest end of the ANCA-positive spectrum: self-limited cutaneous vasculitis and arthralgias, infection-triggered and without organ damage, consistent with BPI-ANCA pathophysiology. The absence of biopsy confirmation, spontaneous ANCA negativization, and 48-month disease-free follow-up without maintenance immunosuppression are collectively more consistent with a post-infectious immune phenomenon than with primary systemic vasculitis.

Case 3 illustrates the diagnostic challenge of isolated MPO-ANCA positivity without organ involvement. Initial treatment with methotrexate served a dual purpose: managing active arthritis while observing for any emerging vasculitic features. The sustained absence of systemic progression across 24 months, combined with response to adalimumab—a TNF-inhibitor without established efficacy in AAV—and ANCA negativization on biologic therapy, ultimately supported seronegative JIA with incidental ANCA positivity as the definitive diagnosis. Similar situations have been reported in several publications, where, out of a group of 31 patients, 14 had an atypical pattern of pANCA [38]. In a study performed in the same geographical region as our country, it was observed that 20% of rheumatoid arthritis patients included in the study had positive pANCA, suggesting that ANCA positivity in rheumatologic conditions may not always indicate vasculitis.

### Study Limitations

This study has several limitations. First, the retrospective design and small sample size limit the generalizability of our findings. Second, this is a single-center experience, which may not reflect the broader spectrum of ANCA-associated conditions in other pediatric populations. Third, tissue biopsy was not obtained in all cases (particularly Case 2 and Case 3), which would have provided additional histopathological evidence to support or refute the diagnosis of vasculitis. In Case 2, the absence of a biopsy limits the certainty of the BPI-ANCA vasculitis diagnosis, and a post-infectious reactive phenomenon cannot be excluded. In Case 3, biopsy refusal by the parents precluded definitive histopathological differentiation between MPO-ANCA vasculitis and juvenile idiopathic arthritis. Fourth, long-term follow-up data are still being collected, and the ultimate outcomes of these patients remain to be fully determined.

## 5. Conclusions

Collectively, these three cases illustrate that ANCA positivity in children defines a clinical spectrum: from life-threatening multi-organ vasculitis (Case 1) to self-limited post-infectious phenomena (Case 2) and autoimmune arthritis with incidental ANCA seropositivity (Case 3). Accurate diagnosis requires integration of serology, organ assessment, and—wherever feasible—histopathology. Treatment intensity must be individually calibrated to disease severity, given the substantial toxicity of aggressive immunosuppression. Multidisciplinary input is essential in severe disease. Future research should prioritize biomarkers capable of distinguishing true AAV from ANCA-positive mimics and international prospective registries to better characterize long-term pediatric outcomes.

In conclusion, ANCA-positive conditions in children require individualized, organ-matched therapeutic strategies and sustained multidisciplinary follow-up.

## Figures and Tables

**Table 1:** Staged biological investigations according to evolution and treatment.

1. Onset: initiation of MTX + PRED treatment		
HLA-B27—negative	CRP = 15.1 mg/L	Proteinuria-600 mg/24 h
ANA—negative	ESR = 72 mm/h	Microscopic hematuria
Cyclic citrullinated peptide (CCP)—negative	D-dimer = 618 ng/mL	
ANA Panel extended IgG—negative	fibrinogen = 421 mg/dL	
Antiphospholipid syndrome panel—negative		
ANCA-PR3 antibodies positive result > 200 U/mL		
2. Thrombosis of the basilar vein and renal damage: treatment with Cyclophosphamide and pulse therapy with Methylprednisolone		
positive thrombophilia panel	CRP = 19.92 mg/L	Proteinuria 1831 mg/24 h
	ESR = 72 mm/h	Serum creatinine = 142 µmol/L
	D-dimers = 4194 ng/mL	Serum Urea = 14.37 mmol/L
	Fibrinogen = 440 mg/dL	Microscopic hematuria 104.000 RBC/minute in a volume of 80 mL
3. Pulmonary hemorrhage		
Aspergillus-galactomannan-negative	Hb-6.8 g/dL	Serum creatinine 230 µmol/L
1,3BDglucan-negative	CRP-68 mg/L	Serum urea 14.6 mmol/L
Pneumocystis J. DNA-negative	ESR-140 mm/h	
	DDimeri-2875 ng/mL	
4. Worsening with Rituximab		
anti-PR3 IgG = positive (140,3 U/mL)	CRP-52 mg/L	Proteinuria 4252 mg/24 h
	ESR-128 mm/h	Serum creatinine 280 µmol/L
	D-dimers = 3800 ng/mL	Serum urea 16.6 mmol/L
	Fibrinogen = 640 mg/dL	
5. After Immunoglobulin treatment		
anti-PR3 IgG = positive (7 U/mL) normal values < 3 U/mL	CRP-5 mg/L	Proteinuria 2300 mg/24 h
	ESR-18 mm/h	Serum creatinine 120 µmol/L
	D-dimers = 540 ng/mL	Serum urea 8 mmol/L
	Fibrinogen = 234 mg/dL	

**Table 2:** Comparative analysis of clinical and therapeutic characteristics among the three cases.

Characteristic	Case 1 (PR3-ANCA+)	Case 2 (BPI-ANCA+)	Case 3 (MPO-ANCA+)
Age at onset	15 years	12 years	13 years
Sex	Female	Female	Female
Initial presentation	Sinusitis, dysuria, arthralgia	Purpuric lesions, joint swelling	Polyarticular arthritis
ANCA type	PR3-positive	BPI-positive	MPO-positive (1:320)
Major organ involvement	Lungs, heart, kidneys, vessels	None	None
Complications	Pulmonary hemorrhage, pericarditis, thrombosis, CKD 3a	Recurrent purpura with infections	None
Initial treatment	Prednisone + methotrexate	Prednisone	Prednisone + methotrexate
Final treatment	Mycophenolate + rituximab + low-dose Prednisone	Prednisone (tapered)	Adalimumab
Final diagnosis	GPA (PR3-ANCA)	BPI-ANCA vasculitis	Seronegative JIA
Outcome	CKD 3a	Complete remission	Complete remission
Follow-up duration	24 months	48 months	24 months
ANCA evolution over time	PR3-ANCA > 200 U/mL at onset; 140.3 U/mL after rituximab cycle 4; 7 U/mL after IVIG; 7.2 U/mL at last follow-up (persistently elevated)	BPI-ANCA positive at onset; negative at 4 months; transiently re-positive during respiratory infections	MPO-ANCA 1:320 at onset; negative after 3 months of adalimumab; remained negative at all subsequent evaluations

## Data Availability

The data are not publicly available due to privacy constraints.
